# Molecular replacement for small-molecule crystal structure determination from X-ray and electron diffraction data with reduced resolution

**DOI:** 10.1107/S2053273323008458

**Published:** 2023-10-19

**Authors:** Tatiana E. Gorelik, Peer Lukat, Christian Kleeberg, Wulf Blankenfeldt, Rolf Mueller

**Affiliations:** a Department of Structure and Function of Proteins, Helmholtz Centre for Infection Research, Inhoffenstraße 7, Braunschweig, 38124, Germany; b Helmholtz Centre for Infection Research and Department of Pharmacy at Saarland University, Helmholtz Institute for Pharmaceutical Research Saarland, Universitätscampus E8 1, Saarbrücken, 66123, Germany; c Institute for Inorganic and Analytical Chemistry, Technical University of Braunschweig, Hagenring 30, Braunschweig, 38106, Germany; d Institute for Biochemistry, Biotechnology and Bioinformatics, Technical University of Braunschweig, Spielmannstrasse 7, Braunschweig, 38106, Germany; Czech Academy of Sciences, Czech Republic

**Keywords:** electron crystallography, small molecules, molecular replacement

## Abstract

The resolution of 3D electron diffraction (ED) data of small-molecule crystals is often relatively poor, due to either electron-beam radiation damage during data collection or poor crystallinity of the material. Direct methods, used as standard for crystal structure determination, are not applicable when the data resolution falls below the commonly accepted limit of 1.2 Å. Therefore an evaluation was carried out of the performance of molecular replacement (MR) procedures, regularly used for protein structure determination, for structure analysis of small-molecule crystal structures from 3D ED data. In the course of this study, two crystal structures of Bi-3812, a highly potent inhibitor of the oncogenic transcription factor BCL6, were determined: the structure of α-Bi-3812 was determined from single-crystal X-ray data, the structure of β-Bi-3812 from 3D ED data, using direct methods in both cases. These data were subsequently used for MR with different data types, varying the data resolution limit (1, 1.5 and 2 Å) and by using search models consisting of connected or disconnected fragments of BI-3812. MR was successful with 3D ED data at 2 Å resolution using a search model that represented 74% of the complete molecule.

## Abstract 2

Molecular replacement as implemented in *Phaser* was applied to structure analysis of small-molecule single-crystal X-ray and electron diffraction data.

## 1. Introduction

Within the last decade, electron diffraction has developed into a reliable method of structure analysis (Gemmi *et al.*, 2019 ▸), featuring the determination of hydrogen positions (Palatinus *et al.*, 2017 ▸) and absolute configuration (Brázda *et al.*, 2019 ▸). Crystals of small molecules are usually well ordered and diffract well. Consequently, small-molecule crystal structures are traditionally solved by direct methods (Sheldrick, 2008 ▸), which rely on the atomicity condition, setting a strict common requirement on the experimental data, namely that the resolution has to be at least 1.2 Å (Gilmore, 2000 ▸).

In many cases, nano-crystalline materials do not provide sufficiently high resolution diffraction data, whether due to poor crystallinity or radiation damage during data collection. In these situations, direct-space global optimization methods, typically simulated annealing (SA), are employed (Andrusenko *et al.*, 2021 ▸; Lightowler *et al.*, 2022 ▸). SA requires a complete molecule, the position and orientation of which within the unit cell are varied in accordance with the symmetry, until an acceptable agreement between the experimental and calculated diffraction data is reached. Apart from three translational and three rotational degrees of freedom, a scan for torsional degrees of freedom can be added to the global optimization search, as implemented, for instance, in *SIR* (Burla *et al.*, 2015 ▸) and *TOPAS* (Coelho, 2018 ▸). SA methods are relatively robust to low data resolution, making use of additional constraints and restraints provided by rigid fragments and molecular connectivity.

With the expansion of the application field of electron diffraction to natural products (Kim *et al.*, 2021 ▸; Park *et al.*, 2022 ▸; Gorelik *et al.*, 2022 ▸), notorious problems associated with poor crystallinity of the materials and high radiation sensitivity are faced. Consequently, the resolution of electron diffraction data in these cases often does not allow for a traditional approach using direct methods. SA cannot be employed either, as in most cases molecules are very flexible because they possess many torsional degrees of freedom as well as chiral centres that have to be handled individually.

Searching for an alternative phasing method able to handle low-resolution data, we explored employing molecular replacement (MR) for small-molecule X-ray and electron diffraction (ED) data. MR is typically used for structure determination of proteins, notoriously producing non-atomic resolution diffraction data, so we decided to apply MR as it is implemented in *Phaser* (McCoy *et al.*, 2007 ▸) to X-ray and ED data of small molecules.

MR is the commonly used approach for phasing of macromolecular crystallography data (Evans & McCoy, 2008 ▸). The procedure requires a search fragment, which is usually a part or all of the molecule in a known configuration. Usually, a search fragment representing a highly similar part of a known structure is used. Alternatively, secondary and tertiary structure fragments extracted from known structures or distant homologues can be used, as is done in *ARCIMBOLDO* (Millán *et al.*, 2015 ▸), or generated from short sequence libraries (Das & Baker, 2008 ▸). The orientation and position of the fragment are determined subsequently by searching for the maxima of a rotation and translation function constructed from the structure-factor amplitudes of the diffraction data measurement of the new structure and an inverse Fourier transformation of the search fragment placed in a virtual crystal, respectively. Once the optimal position of the search fragment is found, the calculated phases of the structure factors are combined with experimentally measured amplitudes and the scattering density map is calculated. In a successful run, the scattering map envelops the search fragment and introduces additional density in the missing parts of the structure. Thus, MR is in principle a hybrid-space algorithm, first placing the fragment in direct space, and then making use of the inverse Fourier transformation to recalculate the scattering density (electron density for X-rays).

Remarkably, MR was initially developed for small molecules. The pioneering work on the Faltmolekülmethode was presented by Walter Hoppe (1957 ▸), demonstrating the structure determination of phenanthrene­quinone. A few years later, Rossmann & Blow (1962 ▸) employed a similar approach for the structural analysis of a protein. In the following years, MR was rapidly overtaken by macromolecular crystallographers, while the structural analysis of small molecules predominantly relied on direct methods. As a result, MR became closely associated with protein research and was disregarded by small-molecule crystallographers. A lone report on the use of MR for the structural analysis of a small-molecule material from X-ray diffraction data emerged in 2014 (Wierzbicki *et al.*, 2014 ▸). Recently, a report detailing the structure determination of short peptides from ED data was published (Richards *et al.*, 2023 ▸). This report employed search fragments extracted from the Protein Data Bank (https://www.rcsb.org/). To date, no MR studies of small molecules, particularly those of non-amino-acid nature and using ED data, have been documented.

We therefore decided to assess the potential of MR structure analysis of small molecules using ED data. To establish a reference for evaluating the performance of ‘ideal’ data, we also incorporated X-ray data into our analysis.

**Figure 1:**
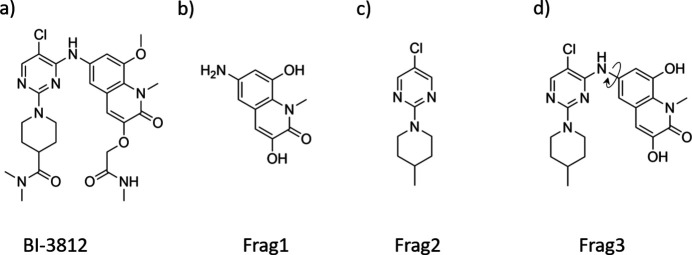
Molecular structure of BI-3812 (*a*), two rigid fragments of the molecule used for MR structure analysis – Frag1 (*b*) and Frag2 (*c*), and Frag3 (*d*) containing one torsion angle.

As a test molecule we selected BI-3812 [Fig. 1 ▸(*a*)]. The compound is a high-potency (IC_50_ ≤ 3 n*M*) inhibitor of the oncogenic transcription factor BCL6 (Kerres *et al.*, 2017 ▸), which is a well known oncogenic driver in *e.g.* diffuse large B-cell lymphoma. The commercially available inhibitor acts by binding to the BTB-domain of BCL6 and thus interferes in the interaction of BCL6 with its co-repressor proteins.

The BI-3812 molecule is particularly attractive for an MR study due to its geometry – the molecule contains flexible side groups [Fig. 1 ▸(*a*)], two rigid fragments [Figs. 1 ▸(*b*), 1 ▸(*c*)] and a single torsional angle between these fragments, which essentially defines the shape of the molecule [Fig. 1 ▸(*d*)]. Using different parts of the molecule as a search fragment, we explored whether the MR procedure would be able to place the fragment correctly, and whether the rest of the unit-cell content would develop within the scattering density map after a successful run.

## 2. Materials and methods

A dry BI-3812 {1-[5-chloro-4-({8-meth­oxy-1-methyl-3-[2-(methyl­amino)-2-oxo­eth­oxy]-2-oxo-1,2-di­hydro­quinolin-6-yl}amino)­pyrimidin-2-yl]-*N*,*N*-di­methyl­piperidine-4-carboxamide} powder sample was obtained from Boehringer Ingelheim within the framework of the opnMe project (https://www.opnme.com/).

### 2.1. Crystal structure of α-BI-3812, X-ray diffraction data

The crystal structure of the α phase was solved from single-crystal synchrotron X-ray diffraction data. Crystallization trials were set up at room temperature with a Crystal Gryphon crystallization robot (Art Robbins Instruments) in Intelli 96-3 plates (Art Robbins Instruments) with 200 nl BI-3812 ethanol solution and 200 nl reservoir solution. Crystals (Fig. S1 in the supporting information) were obtained in several conditions of the Index sparse-matrix crystallization screen (Hampton Research). For data collection, a crystal was harvested from the E6 well, cryo-protected with 10%(*v*/*v*) (*R*,*R*)-2,3-butane­diol and flash-cooled in liquid N_2_.

Data collection was carried out at 100 K on beamline P11 optimized for macromolecular crystallography (Meents *et al.*, 2013 ▸; Burkhardt *et al.*, 2016 ▸) of the PETRA III storage ring at the Deutsches Elektronen-Synchrotron (DESY, Hamburg, Germany). The obtained maximal resolution was limited to 0.66 Å by the detector edge at the closest possible distance to the sample at the shortest reachable wavelength of 0.62 Å (20 keV) of the beamline optics. Diffraction data were initially processed using the *autoPROC* toolbox (Vonrhein *et al.*, 2011 ▸) (Global Phasing) automatically executing *XDS* (Kabsch, 2010 ▸), *Pointless* (Evans, 2006 ▸) and *Aimless* (Evans & Murshudov, 2013 ▸). The XDS_ASCII.HKL file from the integration step of this pipeline was used for further processing and structure solution with *SHELXD* (Sheldrick, 2008 ▸) within the *Olex2* (Dolomanov *et al.*, 2009 ▸) environment. The structure was refined with *SHELXL* (Sheldrick, 2015 ▸). The details of the structure analysis can be found in Table 1 ▸. The CSD (Cambridge Structural Database) deposition number is 2235391.

### 2.2. Crystal structure of β-BI-3812, electron diffraction data

The crystal structure of the β phase was solved from ED data. BI-3812 was recrystallized from ethanol and dispersed in *n*-hexane. A drop of the suspension was placed onto an amorphous carbon-coated transmission electron microscope grid and the excess liquid was removed with filter paper. The grids were dried in air.

Dry grids were clipped into grid holding rings and transferred into a Glacios transmission electron microscope (Thermo Fisher) at room temperature. The sample was cooled to liquid-nitro­gen temperature within the transmission electron microscope. Measurements were performed using the EPU-D (Thermo Fisher) module in continuous-rotation mode (Nederlof *et al.*, 2013 ▸; Nannenga *et al.*, 2014 ▸). Tilt series were collected within ±60° goniometer tilt range with 1° frame interval and a rotation speed of 1° s^−1^. The data were collected using an electron dose rate of 6.016 e nm^−2^ s^−1^. For a full data set consisting of 130 frames, the total dose is then 7.8 e Å^−2^ s^−1^. The original tilt series in MRC format has been deposited at Zenodo (https://doi.org/10.5281/zenodo.7794779). Crystals had a lateral size of a few microns (Fig. S2); the electron beam size used for diffraction data collection was 1.2 µm.

The data reduction was performed with *PETS2* (Palatinus *et al.*, 2019 ▸). The unit cell is triclinic (Table 1 ▸), and no higher symmetry could be deduced from the cell metric, nor observed within the main sections of reciprocal space (Fig. S3). The crystals showed relatively high mosaicity (Fig. S3) and diffuse scattering was observed often.

The expected molecular volume (Hofmann, 2002 ▸) of one BI-3812 molecule is 688.53 Å^3^. The volume of two molecules (2 × 688.53 Å^3^ = 1377.06 Å^3^) is close to the experimentally observed unit-cell volume of 1305.7 Å^3^. Thus, it was reasonable to suggest that the unit cell contains two molecules with no solvent present in the crystal structure.

More than 40 ED tilt series were collected and 15 were selected for further processing. All processed data sets showed the same unit-cell metrics and produced essentially the same structure model. For the final structure analysis, we selected the best-performing data set with all atoms obtained in the structure solution run. We used a data set from a single tilt series without merging.

The structure was solved in *P* 1 with *SHELXD* (Sheldrick, 2008 ▸) and was isotropically refined with *SHELXL* (Sheldrick, 2015 ▸) within the *Olex2* (Dolomanov *et al.*, 2009 ▸) framework, using electron atomic scattering factors (Prince, 2004 ▸). SADI and FLAT restraints were applied during the refinement procedure and electron scattering amplitudes were parametrized as described in the supporting information. The CSD deposition number of the structure is 2301931.

### 2.3. Molecular replacement

For MR, data reduction was performed with *XDS* (Kabsch, 2010 ▸). The ED data set initially stored in MRC format was converted to SMV format with the MRC2SMV_BS.exe converter (Zenodo, https://doi.org/10.5281/zenodo.7322800), which has a built-in pedestal of 10 counts in order to suppress clipping of negative values to zero.

MR was performed with *Phaser* (McCoy *et al.*, 2007 ▸). Electron scattering factors were applied and all other parameters were left at their default values. The search was performed in the *P* 1 (No. 2) space group.

There are several quantitative measures to indicate the success of an MR structure determination run (Oeffner *et al.*, 2018 ▸). In contrast to small-molecule crystallography, where least-squares figures of merit are used, in MR, functions based on maximum log-likelihood gain (*e.g.* LLG) are considered most sensitive for scoring the fragment placement (Read & McCoy, 2016 ▸). The most popular indicator of the success is the square of the translation function *Z*-score (TFZ), which represents the number of standard deviations of the best solution’s LLG over the mean LLG, allowing one to judge whether a solution significantly standing out from alternative arrangements of the search fragment has been found. For protein structures, TFZ values above 8 usually indicate a correctly solved structure, whereas values below 5 mean that that correct solution has not been found.

The MR procedure requires a search fragment that represents a molecule in a conformation closely resembling the expected structure. In the case of macromolecules, homologous structures, domains or structural fragments can be employed as viable search fragments. With the advent of *AlphaFold2* (Jumper *et al.*, 2021 ▸), predicted protein structures have proven to be highly effective as search fragments (Read *et al.*, 2023 ▸).

A scan for different conformations of the search fragment, as done in SA, is not an intrinsic part of MR, but can be created using a set of individual fragments. *Phaser* allows MR runs with multicomponent search, *e.g.* through *exclusive* multicomponent search (i) with different models where the best-fitting model will be selected in a successful run. Alternatively, the search motif can be split into several different fragments, which are then used for an *inclusive* multicomponent search (ii), with the final structure composed of independent individually oriented parts. In principle, there is no limitation on the size and number of fragments used for the inclusive multicomponent search. It has been demonstrated that a set of individual atoms can give an MR structure solution, as long as the experimental data provide a sufficiently high resolution (McCoy *et al.*, 2017 ▸).

The molecular stoichiometry of BI-3812 is C_26_H_32_ClN_7_O_5,_ corresponding to 39 non-hydrogen atoms including a strongly scattering chlorine atom.

The BI-3812 molecule is relatively flexible because of the torsional angles that it contains. We cut off different rigid fragments with defined geometry from the original molecule, and used these as search fragments. Frag1 contains the planar quinoline fragment as shown in Fig. 1 ▸(*b*), with 15 non-hydrogen atoms, representing 38% of the whole molecule. Frag2 contains two rings – pyrimidine and piperidine [Fig. 1 ▸(*c*)] with 14 non-hydrogen atoms (36% of the molecule). Strictly speaking, Frag2 is not rigid, since a restricted rotation is allowed between the pyrimidine and piperidine rings and the piperidine ring can adopt either a chair or boat conformation (Cremer & Pople, 1975 ▸). Frag3 [Fig. 1 ▸(*d*)] is a combination of Frag1 and Frag2 with a torsional degree of freedom between the quinoline and pyrimidine parts containing 29 non-hydrogen atoms (74% of the molecule).

In order to emulate diffraction data with different resolution, we cut off reflections beyond a certain value within the *Phaser* interface. Obviously, this simple approach is a very rough estimate of a real data set with limited resolution, yet it allowed us to obtain general trends and behaviours concerning data resolution effects.

## 3. Results

### 3.1. Crystal structure of α-BI-3812

**Figure 2:**
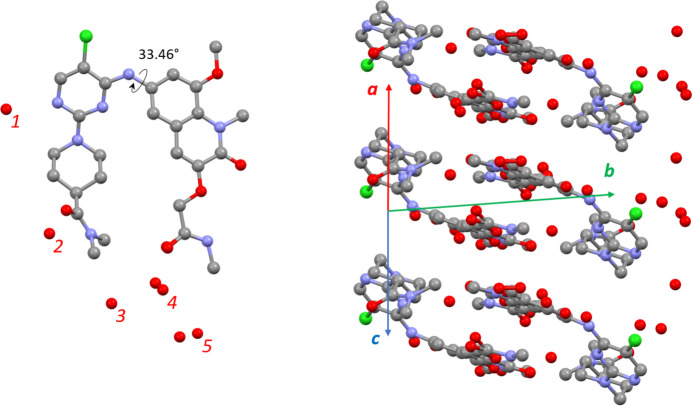
Crystal structure of α-BI-3812 as determined from synchrotron diffraction data: the molecular conformation (left) and the packing of the molecules in the crystal structure viewed along the [101] direction (right). The positions of water molecules 4 and 5 are disordered.

The crystal structure of α-BI-3812 is a pentahydrate (Fig. 2 ▸). Five water molecules are held by hydrogen bonds, and the positions of water molecules 4 and 5 are disordered. The BI-3812 molecule adopts a nearly planar conformation. Notably, the related molecule BI-3802 adopts a non-planar conformation with a torsional angle of 65° (Fig. S5) within a complex with BCL6 (Słabicki *et al.*, 2020 ▸). Thus, the molecular planarity of BI-3812 within the crystal structure is obviously a packing effect; planar molecules can generally adopt a more efficient packing with lower packing energy (Schmidt *et al.*, 2007 ▸). The angle between the quinoline and pyrimidine planes is 29.45°. The torsional angle between the quinoline and pyrimidine is 33.46° (Fig. 2 ▸). In the crystal structure, the planar quinoline groups are stacked in pairs (Fig. 2 ▸).

The piperidine ring adopts a chair conformation (Cremer & Pople, 1975 ▸), and its mean plane is almost in plane with the pyrimidine ring plane. This conformation is observed often, as it allows for the maximum degree of conjugation between the N-atom electron lone pair of the piperidine and the π system of the pyrimidine ring (Brameld *et al.*, 2008 ▸). This co-planarity allows the two rings to be treated as a single rigid fragment, given that the conformation of the piperidine ring (chair) is known. We later used the whole fragment Frag2 as a rigid body in MR runs (Sections 3.3.3 and 3.4.3).

### 3.2. Crystal structure of β-BI-3812

**Figure 3:**
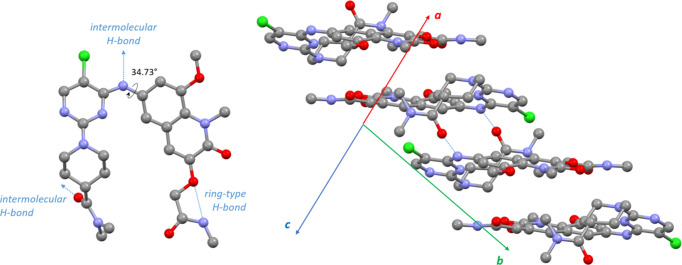
Crystal structure of β-BI-3812 as determined from ED data: the molecular conformation (left) and the packing of the molecules in the crystal structure viewed along the [101] direction (right). Light-blue dotted lines mark the positions of the hydrogen bonds. Hydrogen-bond-connected dimers are stacked as shown on the right.

**Figure 4:**
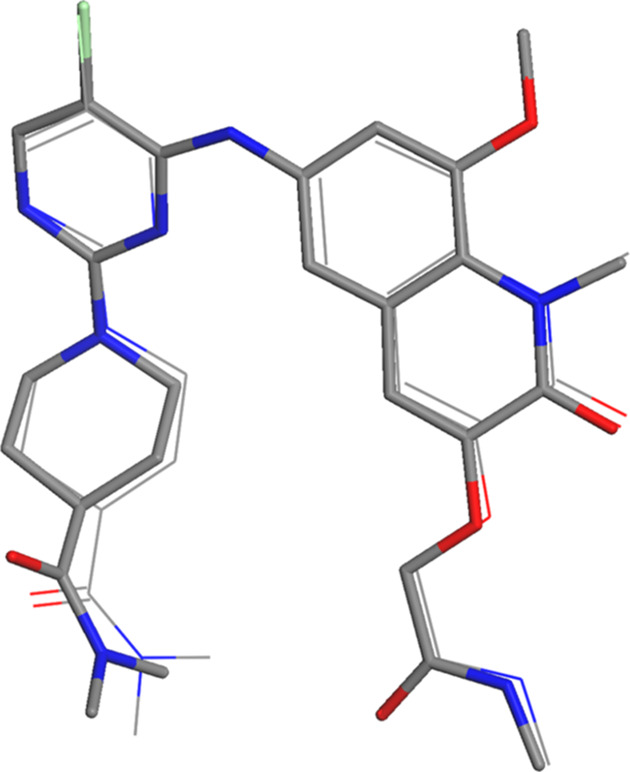
Overlay of the molecular conformations of α-BI-3812 (line representation) and β-BI-3812 (stick representation).

The crystal structure of β-BI-3812 was determined from ED data. In contrast to α-BI-3812, this structure does not contain any solvent molecules. A ring-type intramolecular hydrogen bond is formed, as shown in Fig. 3 ▸. An intermolecular hydrogen bond is formed between amino and carboxyl groups, creating molecular dimers (Fig. 3 ▸). Despite the different crystal habit, the molecular conformation is very similar to that of α-BI-3812. The angle between the quinoline and pyrimidine planes is 31.37° and the torsional angle at the amino group is 34.73°. The piperidine ring is in a chair conformation and in plane with the pyrimidine ring, matching the Frag2 geometry. An overlay of the molecular conformations of the α and β phases is shown in Fig. 4 ▸.

### 3.3. MR of α-BI-3812, X-ray diffraction data

#### 3.3.1. Search fragment – the complete molecule

We first performed MR runs with the whole BI-3812 molecule in the fixed conformation of the α-phase crystal structure. The aim of this run was to place the molecule correctly within the unit cell and identify the five water molecules around it. Three runs were performed with the diffraction data resolution limit set to 1, 1.5 and 2 Å. The TFZ scores of the best solutions were 20.6, 9.9 and 6.9, correspondingly (Table 2 ▸), indicating that the structure was solved correctly in all cases (TFZ > 5).

**Figure 5:**
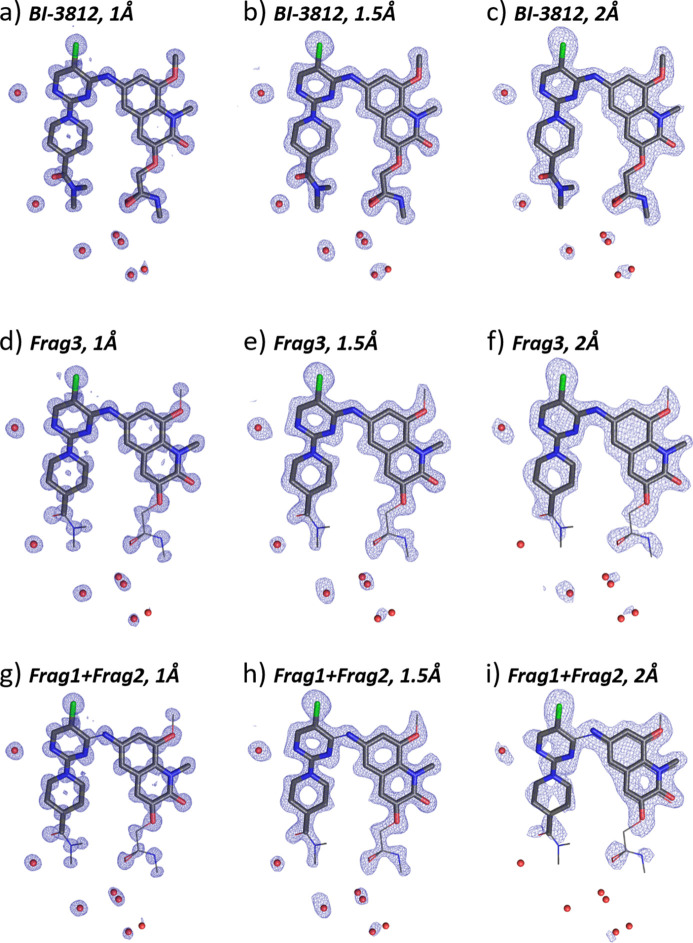
Results of the MR structure solution of α-BI-3812 (X-ray diffraction data) with the complete molecule as the search fragment and data resolution of (*a*) 1.0 Å, (*b*) 1.5 Å, (*c*) 2 Å, Frag3 as the search fragment and the data resolution of (*d*) 1.0 Å, (*e*) 1.5 Å, (*f*) 2 Å, and Frag1 and Frag2 as search fragments and data resolution of (*g*) 1.0 Å, (*h*) 1.5 Å, (*i*) 2 Å. The molecular configuration of the crystal structure (wire representation) is overlaid with the search fragment (bold bonds) and the obtained electron-density map (blue mesh). The 2*mF*_o_ − *DF*_c_ maps are contoured at 1.3σ above the mean, the densities are carved to 2 Å around the selected atoms. The *mF*_o_ − *DF*_c_ maps for (*d*), (*e*) and (*f*) are shown in Fig. S7.

The electron-density maps of all solutions are shown in Figs. 5 ▸(*a*), 5 ▸(*b*), 5 ▸(*c*). The residual density around the molecule corresponded to the positions of water molecules in the structure. The solution with the data resolution limit of 1 Å [Fig. 5 ▸(*a*)] shows clearly resolved atomic positions. These data would also give a solution with direct methods. The resolution limit of 1.5 Å [Fig. 5 ▸(*b*)] lies beyond the classical limit for direct methods, yet the positions of all atoms, also the oxygen atoms of the water molecules, are well placed within the density map. With further deterioration of the data resolution [Fig. 5 ▸(*c*), resolution limit 2 Å], the electron-density map becomes more blurred. Still, electron density envelops all expected atomic positions.

The agreement of the obtained placement of the molecule with the positions of non-hydrogen atoms in the expected crystal structure was quantified by root-mean-square deviations (RMSDs). Only the atoms of the search fragment (the complete BI-3812 molecule, 39 atoms) were used in these calculations. The resulting RMSD values were 0.0094, 0.0287 and 0.0719 Å, respectively, for data resolutions of 1.0, 1.5 and 2.0 Å (Table S2), showing that the correct structure model was found in all cases.

#### 3.3.2. Search fragment: Frag3

We next decided to reduce the search fragment to Frag3, clipping off flexible parts of the molecule and keeping only one torsion angle in the fixed conformation of the structure solution. MR runs with diffraction data clipped at different resolution limits (1.0, 1.5, 2 Å) produced the best solutions with TFZ scores of 14.0, 7.4 and 5.4, respectively, indicating that correct solutions have been found. Indeed, the positions of the fragments within the structure were found correctly [Figs. 5 ▸(*d*), 5 ▸(*e*), 5 ▸(*f*)], and additional electron density around the not-included flexible parts of the molecule as well as around the missing water molecules would allow straightforward inclusion into the model for subsequent refinement steps.

The RMSDs of the Frag3 (29 atoms) placement with respect to the expected positions of the atoms were 0.0101, 0.0649 and 0.1224 Å, respectively, for the data resolutions of 1.0, 1.5 and 2 Å (Table S2).

Notably, even for 2 Å resolution data [Fig. 5 ▸(*f*)], the correct placement of the search fragment was found, and the positions of the atoms adjacent to Frag3 were evident (Fig. S6). A short *SHELX* refinement of the structure with 2 Å data resolution starting from the found placement of Frag3 led to a complete structure (Fig. S7).

#### 3.3.3. Search fragment: Frag1 + Frag2

Up to this point, we have performed a single fragment search, which for Frag3 requires knowledge of the torsion angle between the quinoline and pyrimidine moieties of the compound. If the structure is not known *a priori*, this information would not be available. We therefore decided to run a two-component inclusive search with rigid fragments Frag1 and Frag2 as a search model, which is a more realistic scenario for an unknown crystal structure. Placing of two components into the unit cell increases the number of search parameters and is a more challenging endeavour.

Figs. 5 ▸(*g*), 5 ▸(*h*), 5 ▸(*i*) show the structure solution results for Frag1 and Frag2 as a search model. For all three resolution ranges the fragments were correctly positioned into the structure, matching the conformation of the molecule. The proximity of the amino group of Frag1 to the fourth position of the pyrimidine of Frag2 allowing for bonding is a reasonable criterion for the correct placement of the fragments into the structure. The RMSDs of the Frag1 and Frag2 (29 atoms) placement with respect to the expected positions of the atoms were 0.0254, 0.0770 and 0.2862 Å, respectively, for the data resolutions of 1.0, 1.5 and 2 Å (Table S2). For 2 Å data, the position of Frag1 was slightly off the atomic positions of the molecule [Fig. 5 ▸(*i*)], which was also reflected in the relatively high RMSD value. This shift was immediately corrected by a subsequent refinement procedure (Fig. S8). Thus, despite the low TFZ score of 4.5, the 2 Å data solution can be used as a starting model for the completion of the structure.

We also tried to run MR for the data resolution of 1 Å with Frag1 only (Fig. S9). The BI-3812 molecule contains 39 non-hydrogen atoms; the α-polymorph additionally contains five water molecules, resulting in 44 non-hydrogen atoms in the asymmetric unit. Frag1, consisting of 15 non-hydrogen atoms, represents 34% of the complete structure. Interestingly, Frag1 was placed correctly. Naturally, the electron-density map is more scattered, yet, in principle, the rest of the molecule can be recognized, especially the chlorine atom. Using lower-resolution cut-offs with Frag1 only did not produce any convincing results.

#### 3.3.4. Search fragment: Frag3 with semi-flexible torsion angle

When the torsion angle within Frag3 is unknown, one can separate the fragment into two planar motifs and perform a two-fragment inclusive search (as shown above). Alternatively, one can produce a set of Frag3 conformers with different torsion angles, and perform an exclusive search for the best-fitting conformer. The latter strategy would mimic a model generation for a global optimization procedure as implemented in SA methods for crystallographic analysis.

The torsion angle in Frag3 of the α-BI-3812 structure solution is 33.46°. We created a set of nine Frag3 conformers with different torsion angles in 2° steps (26°, 28°, 30°, 32°, 34°, 36°, 38°, 40°, 42°). The conformer with the torsion angle 34° is the closest to the crystal structure conformation. These nine conformers were used as fragments for exclusive search MR. The TFZ scores of the best solutions were 19.9, 8.4 and 5.7, respectively, for 1.0, 1.5 and 2 Å data resolution. Plots of the LLG and TFZ scores for different torsion angles are shown in Fig. S10.

The torsion angles of the best-fitting conformers were 34°, 36° and 32°, close to the expected value of 33.46°. The conformers with 36° and 32° torsion angle deviate slightly from the expected geometry. This difference will likely be levelled out during subsequent refinement steps. Electron-density maps of the solutions were essentially the same as those for the Frag3 single-component search [Figs. 5 ▸(*d*), 5 ▸(*e*), 5 ▸(*f*)].

An important question is to consider what happens if the correct conformer is not included in the fragment search list. To address this, we generated an additional set of five conformers with torsion angles of 42°, 44°, 46°, 48°, 50°, and ran the MR procedure. The TFZ scores of the solutions were slightly worse than those for the nine conformers search including the correct conformation (Table 2 ▸). In all cases the torsion angle of the best-fitting conformer was 42° – the closest conformation to the structure solution. The electron-density maps also looked similar to those of the Frag3 single-component search [Figs. 5 ▸(*d*), 5 ▸(*e*), 5 ▸(*f*)].

Thus, a manually performed brute-force optimization of the torsion angle is able to deliver a structure model close to the correct structure. The increase of the TFZ scores of solutions for different conformation ranges (Fig. S10) can be used for guiding the search into a certain conformation range, thus optimizing the Frag3 geometry.

### 3.4. Molecular replacement of β-BI-3812, electron diffraction data

#### 3.4.1. Search fragment – the complete molecule

Having analysed the guidelines for structure analysis of α-BI-3812 with MR using X-ray diffraction data, we applied similar procedures to the ED data of β-BI-3812. The major differences between the X-ray and ED data are the unavoidable presence of dynamical scattering and lower completeness (72%) of the ED data. The lower data resolution of the ED data set of 0.9 Å should not influence the procedure, as we only target 1, 1.5 and 2 Å resolution limits. Electron atomic scattering factors were applied; for all other parameters default values were kept.

Our first attempt to run MR with ED data with a full molecule of β-BI-3812 failed for an unexpected reason. The crystal structure of the β phase does not contain any solvent molecules; the BI-3812 molecules are ‘closely packed’. The MR software employed here has been developed for protein crystals and assumes high solvent content (Matthews, 1968 ▸; Kantardjieff & Rupp, 2003 ▸). Apparently, the Matthews coefficient is hard-coded into *Phaser*, such that program runs with solvent-free ultra-compact small-molecule crystal structures are aborted (Read, 2023 ▸).

#### 3.4.2. Search fragment: Frag3

**Figure 6:**
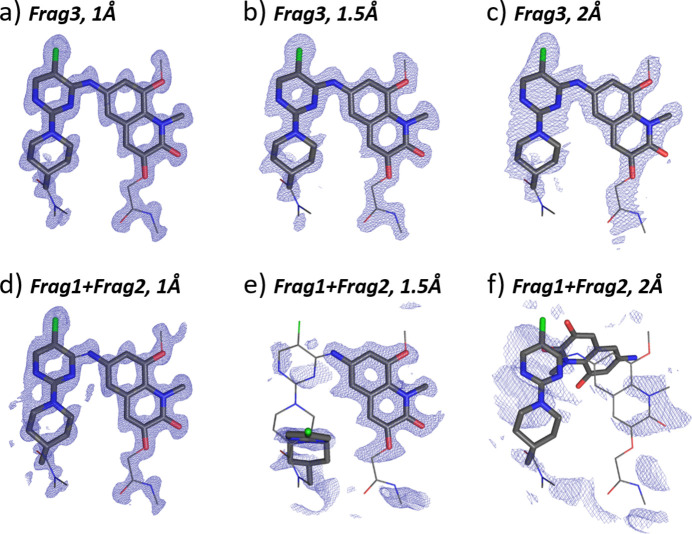
Results of the MR structure solution of β-BI-3812 (ED data) with Frag3 as the search fragment and the data resolution of (*a*) 1.0 Å, (*b*) 1.5 Å, (*c*) 2 Å, and Frag1 and Frag2 as search fragments and data resolution of (*d*) 1.0 Å, (*e*) 1.5 Å, (*f*) 2 Å. The molecular configuration of the crystal structure (wire representation) is overlaid with the search fragment (bold bonds) and the obtained electron-density map (blue mesh). The 2*mF*_o_ − *DF*_c_ maps are contoured at 1.3σ above the mean, the densities are carved to 2 Å around the selected atoms.

Structure solution runs with Frag3 as a search model resulted in solutions with TFZ scores of 13.1, 8.6 and 5.6, respectively, for the data resolution limits of 1, 1.5 and 2 Å (Table 3 ▸). The corresponding scattering density maps are shown in Figs. 6 ▸(*a*), 6 ▸(*b*), 6 ▸(*c*). The diffraction data with a resolution of 1 Å produced a map in which the complete molecule could be unambiguously recognized [Fig. 6 ▸(*a*)]. The reduced resolution of 1.5 Å resulted in a map with well defined Frag3 atomic positions and well resolved flexible parts of the quinoline (Frag1) fragment [Fig. 6 ▸(*b*)]. RMSDs of the Frag3 placement compared with the expected atomic positions were 0.0655, 0.0823 and 0.0887 Å, respectively, for 1, 1.5 and 2 Å data resolution (Table S3), indicating the correct placement of the search fragment.

Interestingly, the data with the poor resolution of 2 Å still delivered a model with the correct placement of Frag3 within the structure [Fig. 6 ▸(*c*)]. Despite the scattering density not looking very sharp, the refinement of the structure delivered the positions of atoms of the flexible fragments (Fig. S9) and completed the molecule.

Encouraged by these results, we moved on to multicomponent searches representing a more practical approach when the torsional angle of the molecule is not known.

#### 3.4.3. Search fragment: Frag1 + Frag2

The TFZ scores of the best solutions for the component search with ED data were 9.4, 3.0 and 3.7, respectively, for ED data of 1, 1.5 and 2 Å resolution. The resulting scattering potential maps are shown in Figs. 6 ▸(*d*), 6 ▸(*e*), 6 ▸(*f*). For 1 Å data resolution, the two fragments were correctly placed into the structure, so that the bond between the amino group of Frag1 and the pyrimidine of Frag2 could easily be recognized. The RMSD of the positioning was 0.2044 Å (Table S3), indicating the correct placement. There is also sufficient scattering potential around the three flexible parts not included in the search models, such that the conformation of the complete molecule can be reconstructed.

An interesting situation is realized for the 1.5 Å resolution data [Fig. 6 ▸(*e*)]. Here, Frag1 was convincingly well surrounded by the scattering potential, so its position indicates that it has been placed correctly. Yet, Frag2 is less clearly defined, no bond can be recognized between the two fragments, and finally Frag1 and Frag2 are not co-planar, as in the determined crystal structure of the β phase. We therefore tried to use the better-defined position of Frag1 as a partial solution and tried to re-search for the position of Frag2. Several iterations improved the final TFZ score, but did not result in the expected structure. We then tried to use the position of Frag1 for the refinement, to see whether the missing atoms would be evident in the Fourier difference map. Yet, the refinement procedure did not converge.

The poor resolution of the 2 Å map [Fig. 6 ▸(*f*)] did not allow for any further structure interpretation.

#### 3.4.4. Search fragment: Frag3 with semi-flexible torsion angle

We finally performed an exclusive multicomponent search for Frag3 using conformers with different values of the torsion angle. We used the same conformer sets as described above for α-BI-3812: a set containing the expected conformation consisting of nine conformers (26°, 28°, 30°, 32°, 34°, 36°, 38°, 40°, 42°), and without the expected conformation, consisting of five conformers (42°, 44°, 46°, 48°, 50°).

**Figure 7:**
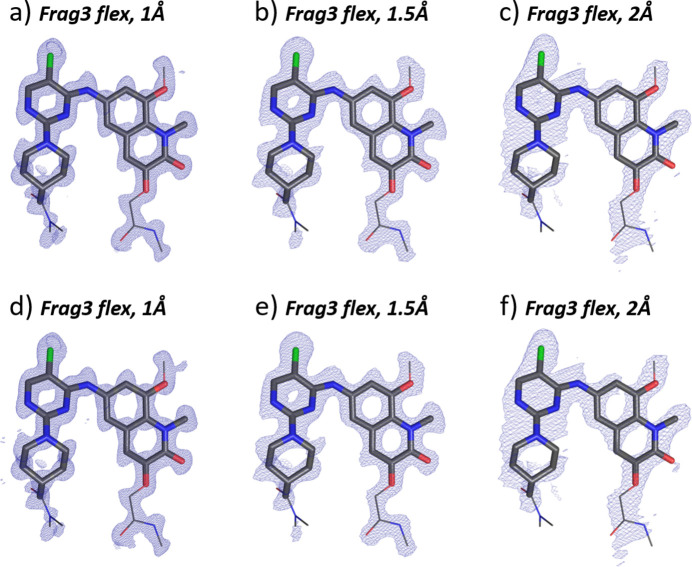
Results of the MR structure solution of β-BI-3812 (ED data) with different conformers of Frag3 as search fragments. In the top row are the scattering potential maps for nine conformers search including the correct geometry (values of torsional angle 26°, 28°, 30°, 32°, 34°, 36°, 38°, 40°, 42°) and ED data resolution of (*a*) 1.0 Å, (*b*) 1.5 Å, (*c*) 2 Å. The bottom row represents obtained scattering potential maps for the five-member torsion conformers set (42°, 44°, 46°, 48°, 50°), without the correct conformation with the data resolution of (*d*) 1.0 Å, (*e*) 1.5 Å, (*f*) 2 Å. The molecular configuration of the crystal structure (wire representation) is overlaid with the selected search fragment (bold bonds) and the obtained electron-density map (blue mesh). The 2*mF*_o_ − *DF*_c_ maps are contoured at 1.3σ above the mean, the densities are carved to 2 Å around the selected atoms.

The torsion angle of Frag3 within the α-BI-3812 crystal structure is 34.37°. For 1 Å data, the best solution had a TFZ score of 13.2 (Table 3 ▸) for the conformer with torsional angle of 36°. The scattering potential map for this solution is shown in Fig. 7 ▸(*a*). Although the closest conformation with 34° was not selected in the best solution, the RMSD of the proposed fragment of 0.0916 Å (Table S3) was very close to the expected atomic positions.

The 1.5 Å data resulted in a solution with a TFZ score of 8.5 and torsional angle of 34° [Fig. 7 ▸(*b*)] with the RMSD of the atoms of 0.1030 Å (Table S3). The 2 Å data produced a solution with a TFZ of 5.5 and 36° torsion angle [Fig. 7 ▸(*c*)], with the RMSD of 0.1149 Å.

All solutions with the five-member conformer set (Table 3 ▸) found the closest conformation with 42°. The corresponding scattering density maps are shown in Figs. 7 ▸(*d*), 7 ▸(*e*), 7 ▸(*f*).

Remarkably, from the set of offered conformers, the procedure was able to select the offered conformer with the torsion angle closest to the conformation realized in the crystal structure (42°). The steep gradient of figures of merit (TFZ, LLG) pushes the solution towards the correct region of torsion angles (Fig. S12). Yet, when conformers are offered with torsion close to the target value, the procedure can hardly identify the correct value within similar score values, as happened for 1 Å data – the 36° solution was picked up instead of the expected 34°. Although the precise value may not be found, the solution obtained should have sufficiently similar geometry, so that a subsequent refinement will correct the molecular geometry.

## 4. Discussion

In order to evaluate the prospects of applying MR procedures for crystal structure determination of small molecules, we determined two crystal structures of BI-3812 from single-crystal X-ray and ED data. The crystal structure of α-BI-3812 – a pentahydrate – was determined from synchrotron data, the crystal structure of β-BI-3812 – an anhydrate – from ED data. The molecular conformation of BI-3812 within the two structures is similar (Fig. 4 ▸).

For both types of data – X-ray and ED – the experimentally obtained data resolution was approximately 1 Å (Table 1 ▸). We performed MR runs with data clipped at different resolutions (1.0, 1.5 and 2 Å) in order to evaluate the applicability of the procedure to experimental data with different crystal quality.

We used X-ray diffraction data and the crystal structure of α-BI-3812 to evaluate the optimal performance of the procedure when applied to a small molecule. ED data of β-BI-3812 represented then a less favourable situation, displaying real problems of ED data associated with the limited completeness and the presence of dynamical scattering effects.

We divided the molecule into several fragments, namely the two small rigid fragments Frag1 and Frag2, and the larger fragment Frag3 containing a single torsion angle. We first tested the procedure with the single rigid fragment Frag3 and then performed several multicomponent searches with Frag1 + Frag2 and Frag3 conformers with different values of the torsion angle. In all cases, X-ray diffraction data performed well and showed the complete structure after the refinement.

The performance of MR with ED data was less compelling. The two-component search with the molecule split into Frag1 and Frag2 only worked for 1 Å data [Fig. 6 ▸(*d*)]. The torsion angle scan represented a more reliable approach, delivering the correct Frag3 conformation even for 2 Å data [Figs. 7 ▸(*c*), 7 ▸(*f*)]. The figures of merit (LLG, TFZ) of solutions with different values of the torsion angle (Fig. S12) allow one to identify the trend and push the solution to the correct geometry. Once the correct value of the torsion angle of Frag3 could be identified, the solution is essentially done by placing Frag3 into the structure (Section 3.4.2). A subsequent refinement would then allow one to complete the missing flexible parts of the molecule (Fig. S11).

Thus, for ED data with 72% completeness and resolution of 2 Å, MR with the search fragment representing 74% of the complete structure (Frag3) was able to deliver a scattering potential map that describes the complete molecule properly [Fig. 6 ▸(*c*)]. Despite the missing flexible parts of the molecule not being apparent within the scattering potential map, the positions of the missing atoms became evident in the course of refinement (Fig. S11). The search for two individual fragments representing 38% (Frag1) and 36% (Frag2) of the complete molecule was only successful with the data resolution of 1 Å. This result is less encouraging as a data resolution of 1 Å typically is sufficient for phasing with direct methods.

The success of MR for ED data is given by a complex balance of data resolution, data completeness (not discussed in this work) and the fraction of the complete structure used as a search fragment. We showed that the exclusive search for an extended fragment with different geometry performed better than the inclusive search for smaller molecule fragments with fixed geometry. We have also demonstrated that a structure determination with 2 Å data is possible, which goes well beyond the direct methods data quality requirements.

For a large and flexible molecule with numerous torsional degrees of freedom, the generation of search fragments with different geometry for MR search can become a very extensive task. In principle, the complete conformational space is sampled at the early stage of SA structure analysis runs. One can think of pipelining the generated conformations into MR. For highly flexible molecules and those containing many stereogenic centres, this task can easily become computationally immense. Any possibility to restrict the conformational space or fix the geometry of certain fragments will help the structure determination. We therefore foresee a significant benefit of combining diffraction data with mass spectrometry or NMR analysis, which very often give a good idea about the principal chemical configuration of the molecule, although details about conformation and stereochemistry may be missing. Obviously, the molecular geometry coming from mass spectrometry or NMR experiments can be different from the geometry in a crystal structure due to the different molecular environment. Yet, firstly, it still can represent a sufficiently ‘good’ initial search model; secondly, we have seen several examples in the field of natural products where the conformation of large molecular fragments is almost entirely preserved in different environments or is similar despite different side groups. The examples are given by chelocardin, having a rigid core fragment (Lešnik *et al.*, 2015 ▸), argyrin, demonstrating practically the same configuration of the polypeptide cyclic part within the crystal structure, in solution and in a protein complex (Gorelik *et al.*, 2022 ▸), and chloro­tonil A and chloro­tonil B, containing different side groups, but preserving the conformation of the macrocycle (Gerth *et al.*, 2008 ▸; Jungmann *et al.*, 2015 ▸; Hofer *et al.*, 2022 ▸). We therefore anticipate that in many cases the geometry of the search fragment can be transferred from a complementary experiment.

## 5. Conclusions

We report two crystal structures of the pharmaceutically relevant compound BI-3812. α-BI-3812 (pentahydrate) was determined from X-ray diffraction data, whereas the structure of β-BI-3812 (anhydrate) was obtained from ED. Both crystal structures were determined with direct methods.

We evaluated the performance of the MR procedure as implemented in *Phaser* for the structure determination of these materials at different data resolution limits. The molecule was split into several rigid fragments that were used as an input model for MR runs. For X-ray diffraction data, reasonable electron scattering density maps were obtained for data with the resolution limit up to 2 Å, using different search strategies. ED data with intrinsically reduced completeness and the presence of dynamical scattering delivered the correct placement of the search fragment for 2 Å resolution data when the search fragment represented 74% of the complete molecule. The positions of missing atoms were evident during the refinement procedure.

Despite the finding that the success of MR for ED data was not as striking as for X-ray data, a step beyond the direct methods imposed limit of 1.2 Å data resolution was evident. We anticipate that the application of the MR technique for ED data of small molecules with sufficiently large rigid fragments or fragments with known conformation will grow in the future.

## Supplementary Material

Crystal structure: contains datablock(s) I, II. DOI: 10.1107/S2053273323008458/lu5029sup1.cif

Structure factors: contains datablock(s) I. DOI: 10.1107/S2053273323008458/lu5029Isup2.hkl

Supporting information. DOI: 10.1107/S2053273323008458/lu5029sup3.pdf

CCDC references: 2235391, 2301931

## Figures and Tables

**Table 1:** Experimental details of the crystal structure determination

	α-BI-3812	β-BI-3812
*Crystal data*		
CSD deposition number	2235391	2301931
Chemical formula	C_26_H_32_ClN_7_O_5_·5(H_2_O)	C_26_H_32_ClN_7_O_5_
*M*_r_	648.11	558.04
Crystal system, space group	Triclinic, *P* 1	Triclinic, *P* 1
Temperature (K)	100	80
*a*, *b*, *c* (Å)	8.948 (2), 12.190 (2), 14.881 (2)	8.4335 (8), 13.4239 (14), 13.6802 (6)
α, β, γ (°)	83.865 (2), 75.600 (3), 80.454 (4)	108.218 (4), 103.749 (9), 107.489 (7)
*V* (Å^3^)	1546.7	1305.7
*Z*, *Z*′	2, 1	2, 1
Radiation type, λ (Å)	Synchrotron, 0.61992	Electrons, 0.0251
μ (mm^−1^)	0.14	–
Crystal size (mm)	0.1×0.1×0.05	0.005×0.001×0.0001†
*Data collection*		
Diffractometer	DESY beamline P11	Glacios transmission electron microscope
No. of measured, independent and observed [*I* > 2σ(*I*)] reflections	55502, 7314, 6940	3313, 1681, 1398
*R*_int_	0.033	0.077
(sin θ/λ)_max_ (Å^−1^), *d*_min_ (Å)	0.753, 0.66	0.7, 1.05
*Refinement*		
*R*_1_[*I* > 2σ(*I*)], *wR*_2_(all data), *S*	0.042, 0.121, 1.04	0.367‡, 0.710, 3.61
No. of reflections in refinement	7314	1681
No. of parameters	480	162
No. of restraints	19	248
Completeness and resolution	90% to *d*_min_ = 0.83 Å, 66% to *d*_min_ = 0.66 Å	72%

† The thickness of the crystal along the incident electron beam is unknown, 100 nm is a very rough estimate.

‡ The refinement was performed with isotropic thermal displacement parameters. Anisotropic refinement resulted in a significantly lower *R*_1_ of 25.51%, yet anisotropic displacement parameters of three atoms turned negative.

**Table 2:** Results of MR solutions for α-BI-3812, X-ray diffraction data TFZ scores for runs with different search fragments and different data resolutions.

	Search fragment				
Data resolution (Å)	Complete molecule	Frag3	Frag1+Frag2	Frag3 nine conformers	Frag3 five conformers
1.0	20.6	14.0	19.4	19.9	16.6
1.5	9.9	7.4	9.0	8.4	7.7
2.0	6.9	5.4	4.5	5.7	5.4

**Table 3:** Results of MR solutions for β-BI-3812, ED data TFZ scores for runs with different search fragments and different data resolutions.

	Search fragment			
Data resolution (Å)	Frag3	Frag1+Frag2	Frag3 nine conformers	Frag3 five conformers
1	13.1	9.4	13.2	11.4
1.5	8.6	3.0, no solution	8.5	7.4
2	5.6	3.7, no solution	5.5	5.2
