# Supplementary material for: Molecular replacement for small-molecule crystal structure determination from X-ray and electron diffraction data with reduced resolution
Source: Acta Crystallogr A Found Adv. 2023 Oct 19;79(Pt 6):504–14. doi: 10.1107/S2053273323008458 (PMC10626656; doi:10.1107/S2053273323008458)
Supplement: Supplementary file 3 [file a-79-00504-sup3.pdf]

FOUNDATIONS ADVANCES

Volume 79 (2023)

Supporting information for article:

Molecular replacement for small-molecule crystal structure determination from X-ray and electron diffraction data with reduced resolution

Tatiana E. Gorelik, Peer Lukat, Christian Kleeberg, Wulf Blankenfeldt and Rolf Mueller

1. Crystals of BI-3812

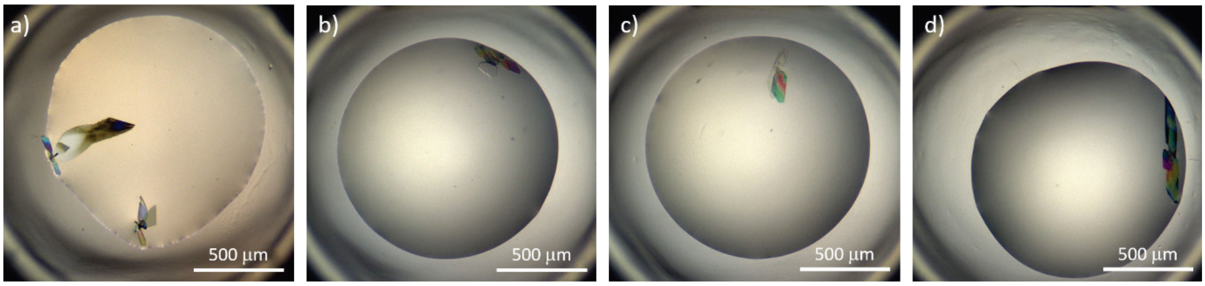

Figure S1 Crystals of **α-BI-3812** grown in a crystallization plate with INDEX screen (Hampton Research). The crystals were grown in B2 (a), E6 (b), E7 (c) and G9 (d) wells. A crystal from E6 well (b) was used for the data collection and structure analysis.

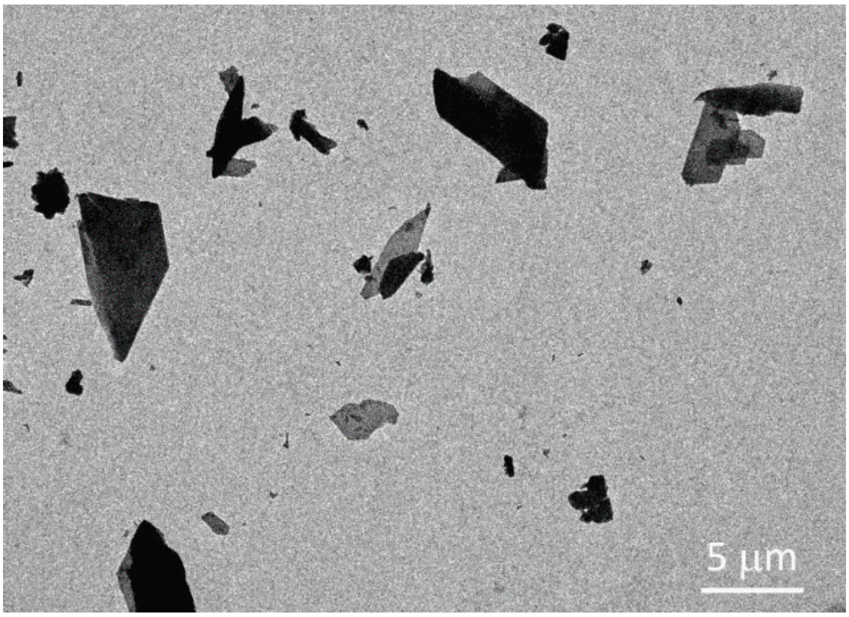

Figure S2. TEM image of crystals of **β-BI-3812**.

2. Electron diffraction data of β-BI-3812

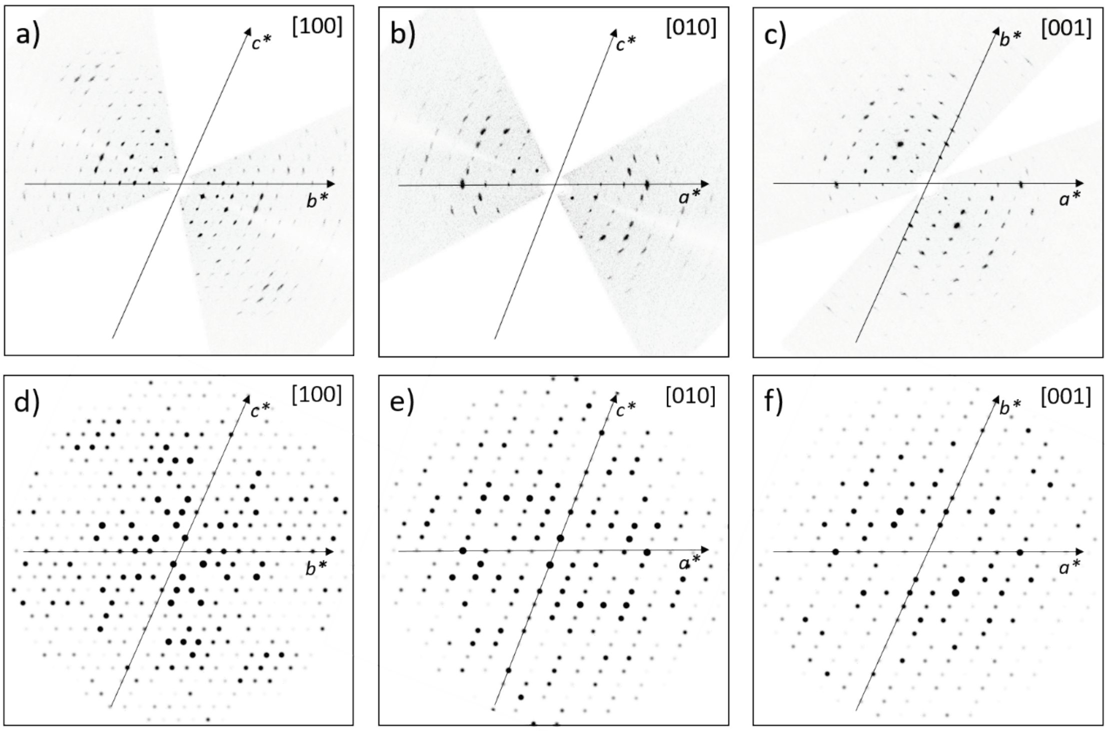

Figure S3. Electron diffraction data of **β-BI-3812**: experimental (a, b, c) and simulated (d, e, f) main sections of the reciprocal space.

3. Atomic scattering amplitudes for electrons

Parametrization for atomic scattering amplitudes for electrons is tabulated with 10 parameters in the form $f_{el}(s) = \sum_{i=1}^{5} a_i \exp(-b_i \cdot s^2)$ (Prince. E. (ed.) International Tables for Crystallography. Vol. C, Mathematical, Physical and Chemical Tables. Kluwer Academic Publishers, 2004, Table 4.3.2.2.), while SFAC command in SHELXL requires 9 parameters of an exponential series in the form $f_{el}(s) = \sum_{i=1}^{4} a_i \exp(-b_i \cdot s^2) + c$. We therefore re-fitted the atomic scattering amplitudes for electrons for hydrogen, carbon, nitrogen, oxygen and chlorine with the 9 parameters ($a_{1\text{-}4}$, $b_{1\text{-}4}$, c). The results of the fitting procedure are presented in Figure S4, the fitted coefficients are given in Table S1.

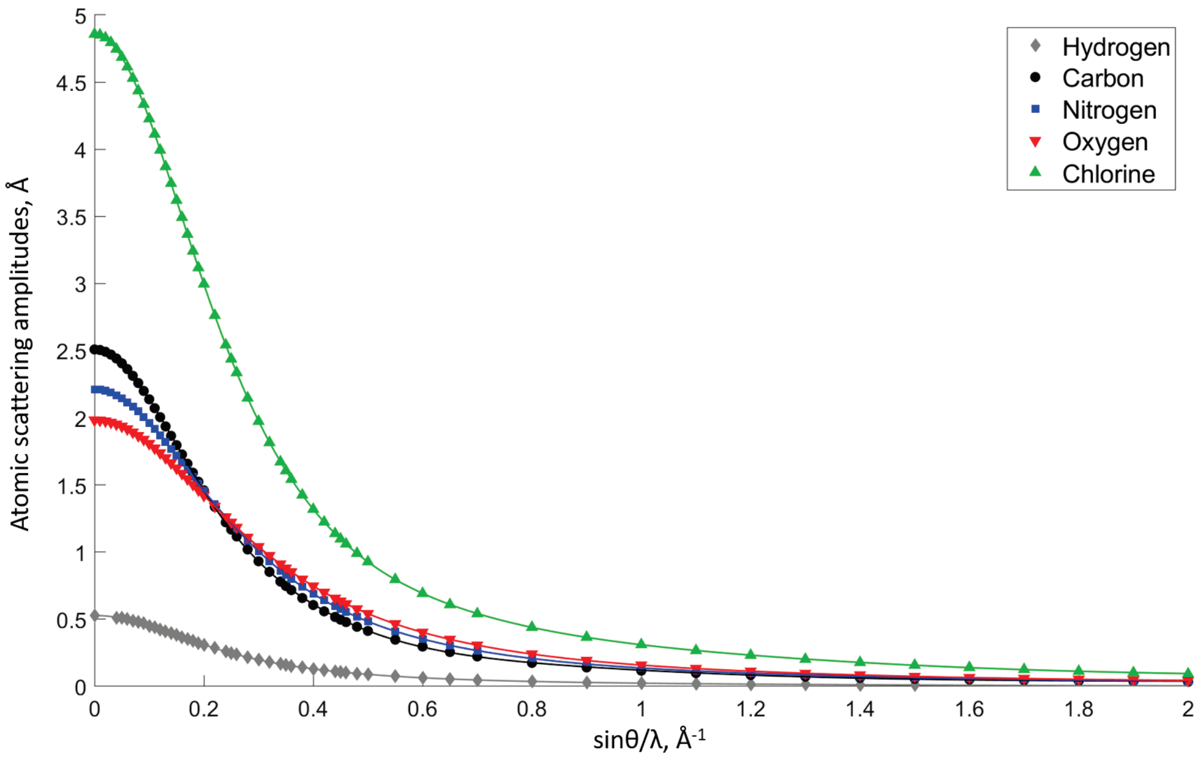

Figure S4. Electron atomic scattering amplitudes for hydrogen (grey), carbon (black), nitrogen (blue), oxygen (red) and chlorine (green). The scatter plots show values for atomic scattering amplitudes as given in the International Tables for Crystallography vol C (Prince. E. (ed.) International Tables for Crystallography. Vol. C, Mathematical, Physical and Chemical Tables. Kluwer Academic Publishers, 2004), Table 4.3.1.1, the solid lines are results of the parameterization with 9 coefficients as required for SHELXL SFAC command.

Table S1. parameters of the re-fitted atomic scattering amplitudes for hydrogen, carbon, nitrogen, oxygen and chlorine in the form $f_{el}(s) = \sum_{i=1}^{4} a_i \exp(-b_i \cdot s^2) + c$

	**H**	**C**	**N**	**O**	**Cl**
a1	0.11297	1.0287	0.35695	0.29995	1.0118
a2	0.19127	-1.0784	0.62767	0.61311	1.0557
a3	0.18353	1.3788	0.21632	0.23185	2.2406
a4	0.038325	1.1367	0.97876	0.80347	0.47483
b1	3.8108	30.3816	36.0762	28.3226	36.2392
b2	30.2952	1.2854	3.9898	3.5102	4.1273
b3	11.0503	1.2854	0.85177	0.78833	13.0584
b4	0.66014	7.8895	12.4013	10.2714	0.77165
c	0.001685	0.037306	0.033103	0.034779	0.073685

The SFAC commands used for the SHELXL refinement procedure are listed below:

SFAC H 0.11297 3.8108 0.19127 30.2952 0.18353 11.0503 0.038325 0.66014 0.001685 0 0 0 0.310 1.008

SFAC C 1.0287 30.3816 -1.0784 1.2854 1.3788 1.2854 1.1367 7.8895 0.037306 0 0 0 0.77 12.011

SFAC N 0.35695 36.0762 0.62767 3.9898 0.21632 0.85177 0.97876 12.4013 0.033103 0 0 0 0.75 14.007

SFAC O 0.29995 28.3226 0.61311 3.5102 0.23185 0.78833 0.80347 10.2714 0.034779 0 0 0 0.73 15.999

SFAC CL 1.0118 36.2392 1.0557 4.1273 2.2406 13.0584 0.47483 0.77165 0.073685 0 0 0 0.99 35.453

4. Comparison with BI-3802 conformation within a complex with BCL6

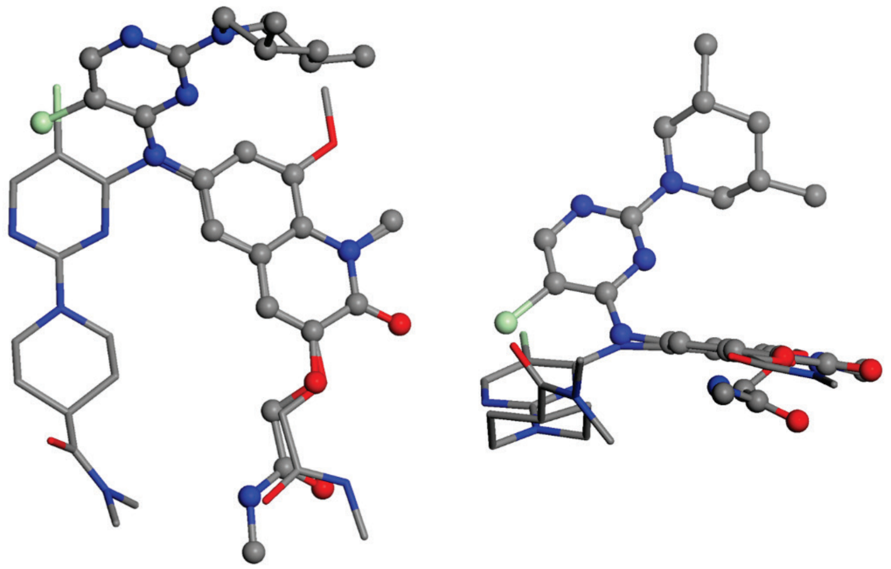

Figure S5. Overlay of **BI-3812** molecule (stick model) in the conformation realized in the α phase with the **BI-3802** (ball and stick model) in the conformation within a complex with BCL6 (Słabicki et al., 2020), view onto the **BI-3812** molecule plane (left side), and along the **BI-3812** molecule plane (right side).

5. Molecular replacement

Table S2. Results of molecular replacement solutions for **α-BI-3812**, X-ray diffraction data. Root Mean Square Deviation (RMSD) of the fragment placement as found by MR compared to the expected crystal structure. Only atoms present in the search fragment were used for the calculation.

	Search fragment				
Data resolution	Complete molecule (39 atoms)	Frag3 (29 atoms)	Frag1+Frag2 (29 atoms)	Frag3 9 conformers	Frag3 5 conformers
1.0 Å	0.0094	0.0101	0.0254	0.0179	0.1585
1.5 Å	0.0287	0.0649	0.0770	0.0546	0.1660
2.0 Å	0.0719	0.1224	0.2862	0.1117	0.1847

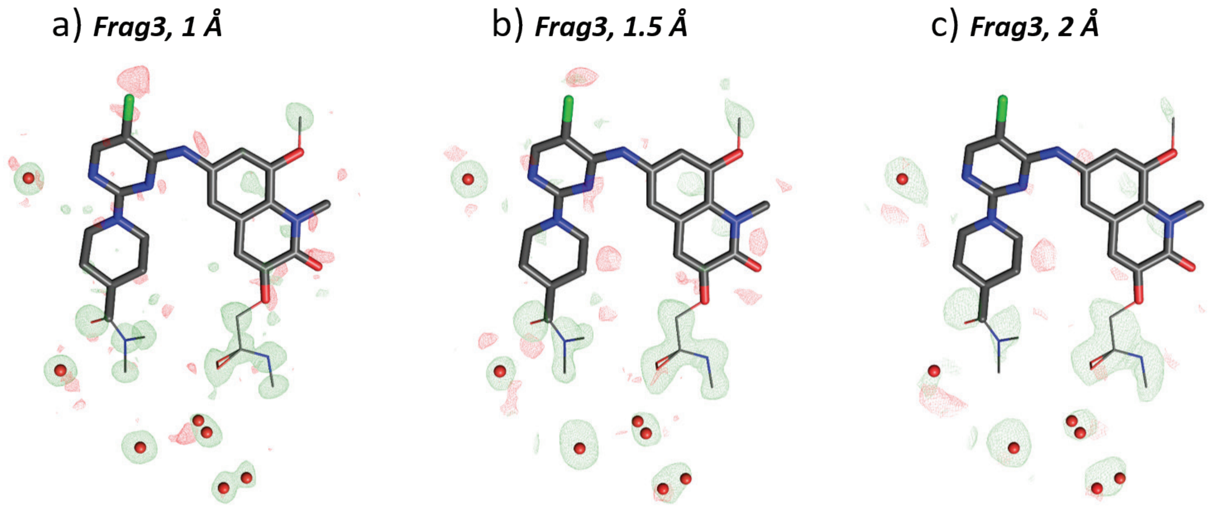

Figure S6 Results of the MR structure solution of **α-BI-3812** (X-ray diffraction data) with **Frag3** as the search fragment and data resolution of a) 1.0 Å, b) 1.5 Å, c) 2 Å. The molecular configuration of the crystal structure (wire representation) is overlaid with the search fragment (bold bonds) and the obtained electron density map The mF_o-DF_c maps are contoured at 2 σ above the mean, the densities are carved to 2 Å around the selected atoms. Red meshes represent negative values, green – positive.

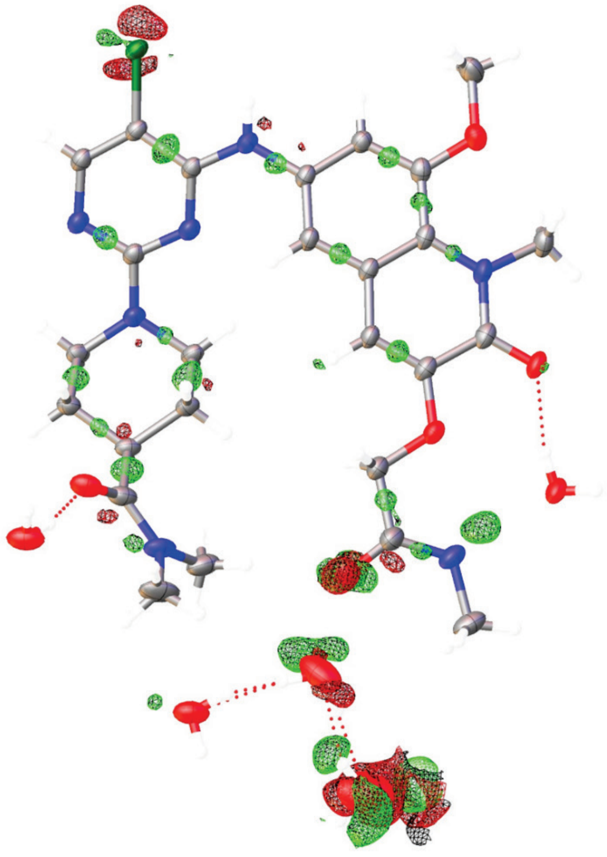

Figure S7 Refinement (SHELXL, in Olex2) of the MR solution of **α-BI-3812** (X-ray diffraction data) with **Frag3** at 2 Å data resolution. R_1 6.40%.

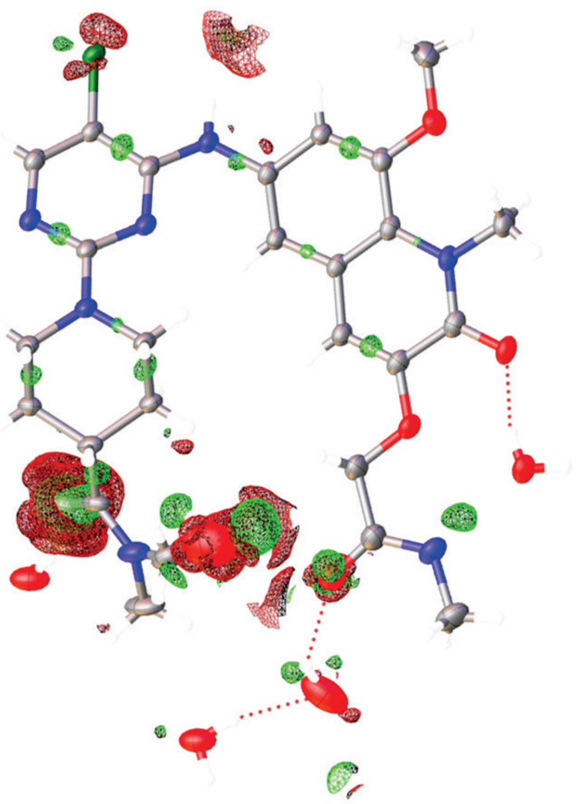

Figure S8 Refinement (SHELXL, in Olex2) of the MR solution of **α-BI-3812** (X-ray diffraction data) with **Frag1** and **Frag2** at 2 Å data resolution. R_1=7.22%.

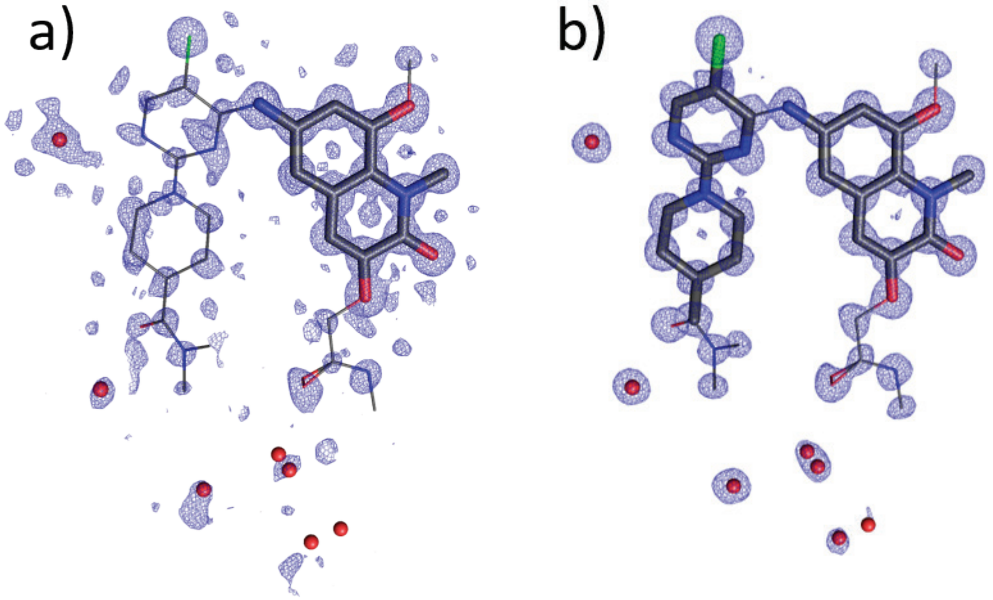

Figure S9 Results of the molecular replacement structure solution of **α-BI-3812** (X-ray diffraction data) with **Frag1** only (a) and data resolution of 1.0 Å (TFZ = 8.8); in (b) the result of MR run with **Frag1** and **Frag2** as search fragments for the same resolution limit of 1.0 Å (this map is already presented in the main text in Figure 5g) is shown for comparison. The 2mF_o-DF_c maps are contoured at 1.3 σ above the mean, the densities are carved to 2 Å around the selected atoms.

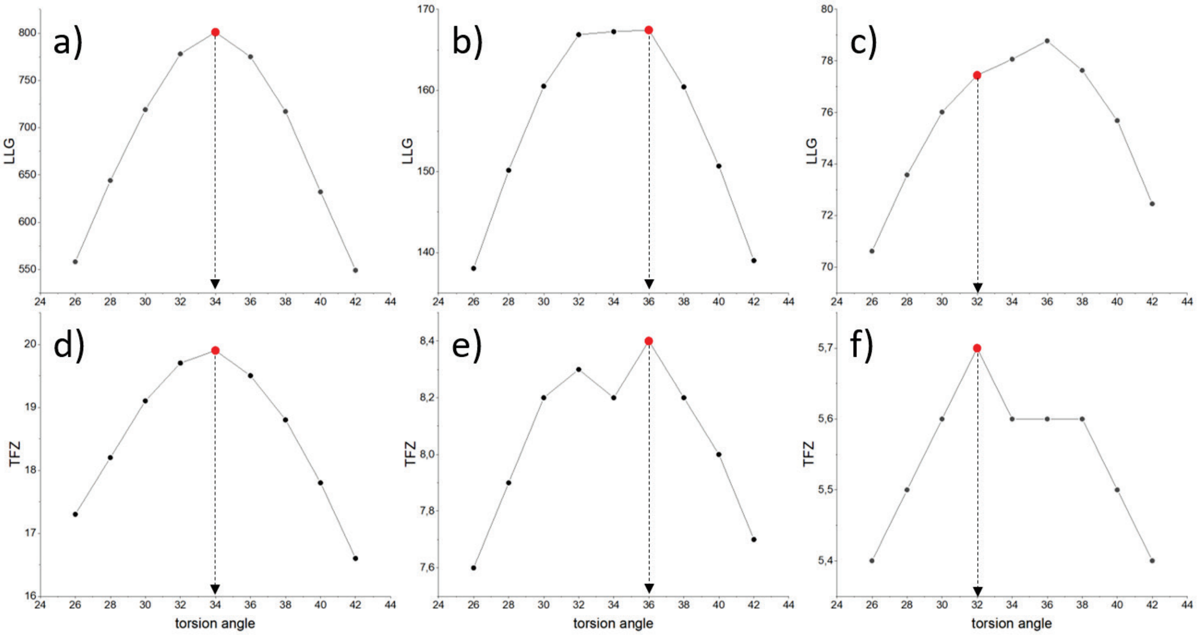

Figure S10 Results of the MR structure solution of **α-BI-3812** (X-ray diffraction data) with **Frag3** with different values of the torsion angle: a) LLG for data resolution 1.0 Å, b) LLG for data resolution 1.5 Å, c) LLG for data resolution 2 Å; d) TFZ for data resolution 1.0 Å, e) TFZ for data resolution, 1.5 Å; f) TFZ for data resolution 2 Å.

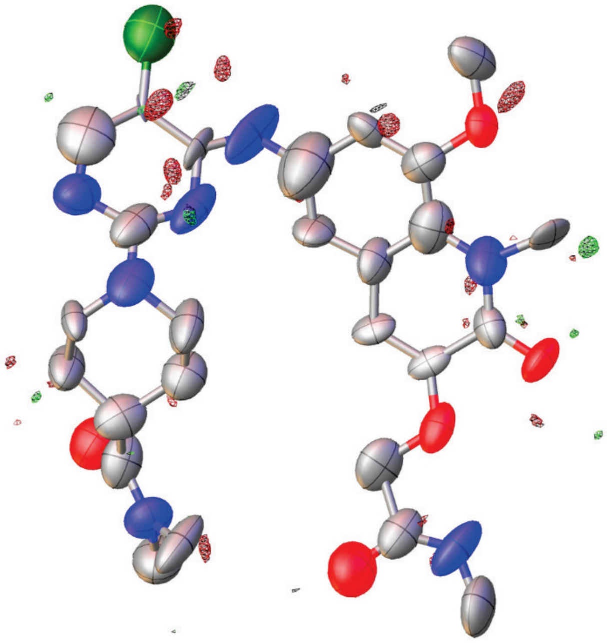

Figure S11 Refinement (SHELXL, in Olex2) of the MR solution of **β -BI-3812** (electron diffraction data) with **Frag3** at 2 Å data resolution. R_1=25.60%.

Table S3. Results of molecular replacement solutions for **β-BI-3812**, electron diffraction data. Root Mean Square Deviation (RMSD) of the fragment placement as found by MR compared to the expected crystal structure. Only atoms present in the search fragment were used for the calculation.

	Search fragment			
Data resolution	Frag3	Frag1+Frag2	Frag3	Frag3

			9 conformers	5 conformers
1 Å	0.0655	0.2044	0.0916	0.1530
1.5 Å	0.0823	-	0.1030	0.1695
2 Å	0.0887	-	0.1149	0.1688

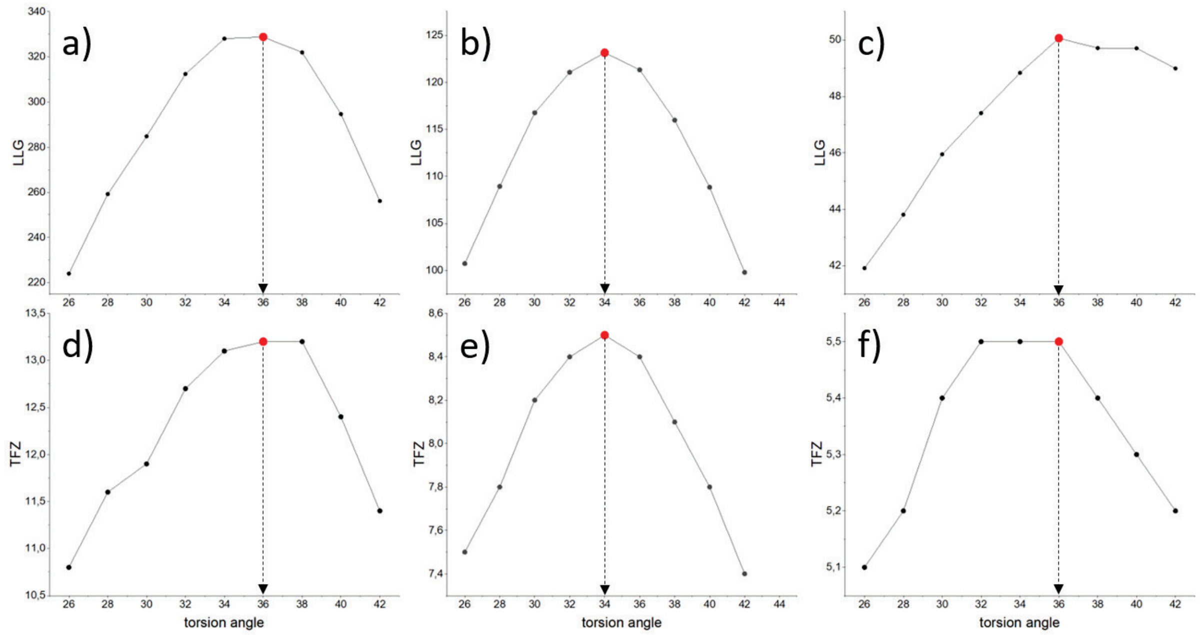

Figure S12 Results of the MR structure solution of **β-BI-3812** (electron diffraction data) with **Frag3** with different values of the torsion angle: a) LLG for data resolution 1.0 Å, b) LLG for data resolution 1.5 Å, c) LLG for data resolution 2 Å; d) TFZ for data resolution 1.0 Å, e) TFZ for data resolution, 1.5 Å; f) TFZ for data resolution 2 Å.
